# Analysis of Quality Differences in Radix Dipsaci before and after Processing with Salt Based on Quantitative Control of HPLC Multi-Indicator Components Combined with Chemometrics

**DOI:** 10.1155/2024/2109127

**Published:** 2024-02-07

**Authors:** Hangsha Wu, Yue Lv, Rui Tang, Mingfang Zhao, Yafei Li, Feiyang Wei, Changyu Li, Weihong Ge, Weifeng Du

**Affiliations:** ^1^School of Pharmaceutical Sciences, Zhejiang Chinese Medical University, Hangzhou 311402, China; ^2^Academy of Chinese Medical Sciences, Zhejiang Chinese Medical University, Hangzhou 310053, China; ^3^Research Center of Traditional Chinese Medicine Processing Technology, Zhejiang Chinese Medical University, Hangzhou 311401, China; ^4^Zhejiang Chinese Medical University Chinese Medicine Yinpian Co., Ltd, Hangzhou 311401, China

## Abstract

Radix Dipsaci (RD) is the dry root of the *Dipsacus asper* Wall. ex DC., which is commonly used for tonifying the kidney and strengthening bone. The purpose of this study was to analyze the difference between raw and salt-processed RD from the chemical composition comprehensively. The fingerprints of raw and salt-processed RD were established by HPLC-DAD to determine the contents of loganin (LN), asperosaponin VI (AVI), caffeic acid (CaA), dipsanoside A (DA), dipsanoside B (DB), chlorogenic acid (CA), loganic acid (LA), isochlorogenic acid A (IA), isochlorogenic acid B (IB), and isochlorogenic acid C (IC). The results showed that after processing with salt, the components with increased contents were LA, CaA, DA, and AVI, and the components with decreased contents were CA, LN, IB, IA, IC, and DB. Then, the chemometric methods such as principal component analysis (PCA) and fisher discriminant analysis (FDA) were used to evaluate the quality of raw and salt-processed RD. In the classification of raw and salt-processed RD, the order of importance of each chemical component was LA > DB > IA > IC > IB > LN > CA > DA > AVI > CaA. These integrated methods successfully assessed the quality of raw and salt-processed RD, which will provide guidance for the development of RD as a clinical medication.

## 1. Introduction

Radix Dipsaci (RD) is the dry root of the *Dipsacus asper* Wall. ex DC., with the effect of tonifying the liver and kidney, strengthening bones and sinews, renewing fractures, and stopping collapse and leakage. The main chemical components of RD are saponins, alkaloids, iridoids, and lignans [1–5]. In clinical practice, RD is commonly used in the treatment of osteoarthritis, osteoporosis, and kidney-yang deficiency [6–10]. At present, the types of decoction pieces used in clinical practice are mainly raw Radix Dipsaci (RRD), wine-processed Radix Dipsaci, and salt-processed Radix Dipsaci (SRD). Among them, SRD is a processing method developed in modern times.

Chemometrics is an emerging interdisciplinary discipline formed by the combination of mathematics, statistics, computer science, and chemistry and is an important means of material-based research of traditional Chinese medicine (TCM). It can introduce multivariate analytical methods into chemical research, process and analyze chemical measurement data in multiple ways, create and optimize various chemical models, and extract the components, structures, and other related information of related substance systems from complex chemical measurement data to the maximum extent. At present, the combination of spectroscopic data and chemometric methods has been used by many scholars to research TCM [11–13]. In this study, HPLC-DAD fingerprints of RD were established, and 10 different components in RD before and after processing with salt were selected for determination and combined with the chemometric methods such as principal component analysis (PCA) and fisher discriminant analysis (FDA) to establish a more comprehensive and quantitative chemical pattern identification and quality evaluation method for the samples of RRD and SRD, providing a certain scientific basis for the later development of studies on spectrum-effect relationship.

## 2. Materials

### 2.1. Instruments

U3000 high-performance liquid chromatograph (Thermo, USA), ME-204E electronic analytical balance (0.01 g, Mettler Toledo, Switzerland), NT-xs105 electronic analytical balance (0.01 mg, Mettler Toledo, Switzerland), and DFT-200 portable high-speed traditional Chinese medicine pulverizer were purchased from Wenling Linda Machinery Co., Ltd., China; KQ-500DB CNC ultrasonic cleaner was purchased from Kunshan Ultrasound Instrument Co., Ltd., China; GDC-750 electromagnetic herbal machine roaster was purchased from Hangzhou Haishan Pharmaceutical Equipment Co., Ltd., China.

### 2.2. Reagents

Loganin (LN, Lot 111640–201808, purity 99%), asperosaponin VI (AVI, Lot 111685–201907, purity 94.3%), caffeic acid (CaA, Lot 110885–201703, purity 99.7%), dipsanoside A (DA, Lot 1647-0025, purity 99%), and dipsanoside B (DB, Lot 1647-0026, purity 99%) were purchased from the National Institutes for Food and Drug Control (Beijing, China). Chlorogenic acid (CA, Lot 120052–201912, purity 98%), loganic acid (LA, Lot 130008–201908, purity 98%), isochlorogenic acid A (IA, Lot 250034-202003, purity 98%), isochlorogenic acid B (IB, Lot 250035-202003, purity 98%), and isochlorogenic acid C (IC, Lot 250036-202003, purity 98%) were purchased from Shanghai Hongyong Biotechnology Co., Ltd. (Shanghai, China).

Methanol (analytical purity) was purchased from Guangdong Guanghua Technology Co., Ltd. (Guangdong, China). Phosphoric acid (analytical purity) was purchased from Zhejiang Hannuo Chemical Technology Co., Ltd. (Zhejiang, China). Acetonitrile (HPLC grade) was purchased from Tedia (Fairfield, USA). The purified water was purchased from Hangzhou Wahaha Group Co., Ltd. (Hangzhou, China).

### 2.3. Sample Collection

RRD was purchased from different origins in China and was authenticated by Professor Weihong Ge (School of Pharmacy, Zhejiang Chinese Medical University). SRD was prepared from RRD. The processing method of SRD was as follows: mixed RRD with salt water evenly, made it moist, stir-fried for 10 minutes at 160°C in a frying pan, took it out, and let it cool (every 100 kg RRD with 2 kg salt). The samples were stored in the herbarium of Zhejiang Chinese Medical University Chinese Medicine Yinpian Co., Ltd. (Hangzhou, China). The sample information is shown in Table 1.

## 3. Methods

The HPLC method for RD was formulated and optimized by our group in a previous work and has been published in other articles by Wu et al. [14]. In this paper, we followed this method for the determination of the content of each component in RD.

### 3.1. Standard and Sample Solution Preparation

The standard solution was prepared in 80% methanol. The concentrations of LA, CA, LN, AVI, CaA, IA, IB, IC, DA, and DB were 953.54, 477.26, 1185.03, 1952.01, 420.73, 830.06, 602.70, 602.70, 110.85, and 40.22 *μ*g/mL.

The sample powder (80 mesh) was weighed 0.5 g accurately and placed in a conical flask with a stopper, and 25 mL of 80% methanol was added and weighed accurately. The solution was ultrasonicated (power 300 W and frequency 50 kHz) for 30 min and then weighed again. 80% methanol was used to make up the lost weight, and the sample solution was obtained by filtering through a 0.45 *μ*m microporous membrane.

### 3.2. HPLC Conditions

The chromatographic column was Agilent Zorbax SB-C_18_ column (4.6 × 250 mm, 5.0 *μ*m); the mobile phase was 0.05% phosphoric acid aqueous solution (A)-acetonitrile (B), gradient elution: 0∼5 min, 2%∼6% B; 5∼18 min, 6%∼10% B; 18∼40 min, 10%∼20% B; 40∼70 min, 20%∼25% B; 70∼80 min, 25%∼35% B; 80∼90 min, 35%∼60% B; 90∼110 min, 60%∼70% B; 110∼120 min, 70% B. Column temperature was 25°C, flow rate was 0.8 mL/min, UV detection wavelength was 215 nm, and the injection volume was 10 *μ*L.

### 3.3. HPLC Methodological Investigation

Linear relationship investigation took the standard solution and diluted it with 80% methanol to make 10 gradient concentration solutions from high to low. According to the chromatographic conditions under “3.2,” injected 10 *μ*L of the different concentration standard solution. Drew a standard curve with peak area (y) and concentration (x, *μ*g/mL) to calculate the regression equation. At the same time, we determined the limit of detection (LOD) of the injection concentration when *S/N* = 3 and the limit of quantification (LOQ) of the injected concentration when *S/N* = 10.

Precision took the same R1 sample solution and continuously injected for 6 times according to “3.2”; repeatability took the R1, prepared 6 samples of the sample solution in parallel, and injected according to “3.2”; stable properties took the R1 sample solution and injected according to “3.2” at 0, 2, 4, 8, 12, 16, and 24 hours, respectively (sample solutions were stored at room temperature). The peak areas of the three investigation items were recorded, respectively, and the relative retention time (RRT) and relative peak area (RPA) RSD of each common peak were calculated with AVI as the reference peak.

The recovery rate of sample addition weighed 6 RD samples powders (R1, S1) with known content, respectively, and added 10 standard substances, respectively (according to the content of the component in the sample, we added a certain volume of corresponding dilutions and then evaporated the solvent). We prepared the tested solution according to “3.1,” injected according to “3.2,” and analyzed it to obtain the average value and RSD of the recovery rates of 10 components.

### 3.4. Establishment of HPLC Fingerprints and Content Determination

We took each batch of RRD and SRD to prepare the sample solution according to “3.1,” injected according to “3.2,” then imported the collected chromatographic data into “Evaluation of Similarity of Chinese Medicine Fingerprints Software,” respectively, generated the contrast map of raw and salt-processed RD, calculated the similarity, pipetted 10 *μ*L of the standard solution to inject it into HPLC, and used the standard data to determine the content of LA, CA, LN, AVI, CaA, IA, IB, IC, DA, and DB in each sample.

### 3.5. Chemometric Analysis

The data of 10 components in each sample obtained by the content determination were imported into SPSS for PCA and FDA, and the difference in quality between the RRD and SRD was analyzed.

## 4. Result and Analysis

### 4.1. Results of Methodological Investigation

The developed method was used to evaluate the linear range, recovery rate, precision, repeatability, and stability of the method for the determination of 10 components.  Table 2 shows that the *r* of 10 components was all greater than 0.999 in the linear range, presenting a good linear relationship, which meets the experimental requirements. The results of precision, repeatability, and stability showed that the RSD of RRT and RPA were both less than 3%, and the similarity was both greater than 0.995, indicating that the method could be used for HPLC detection of RD. The sample recovery rate results in Table 3 showed that the recovery rates of the 10 components in the raw and salt-processed RD were all within the range of 95% to 100%, indicating that the accuracy was good and met the experimental requirements.

### 4.2. HPLC Fingerprints of Samples

**Figure 1:**
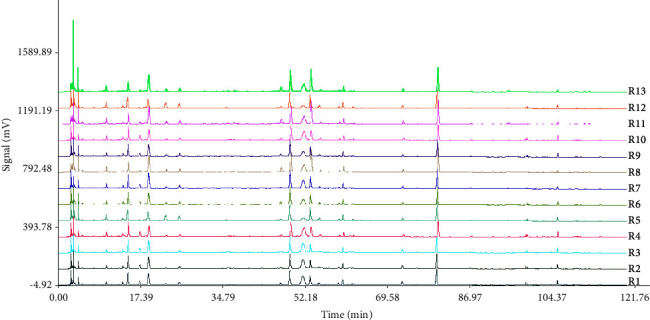
The HPLC fingerprints of 13 batches of RRD.

**Figure 2:**
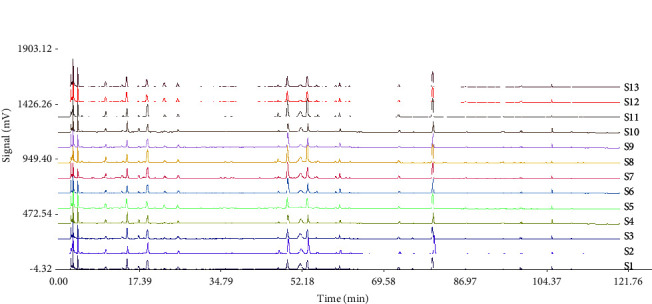
The HPLC fingerprints of 13 batches of SRD.

**Figure 3:**
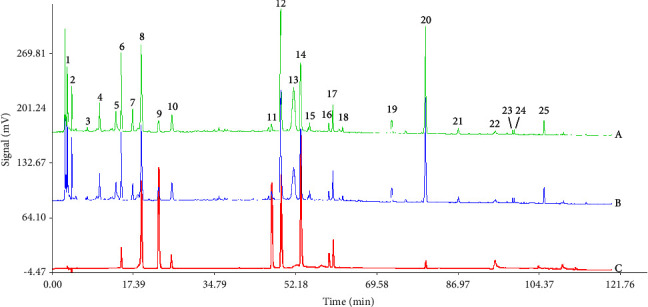
The HPLC spectrogram of RRD (A), SRD (B), and mixed reference (C) (6-LA, 8-CA, 9-CaA, 10-LN, 11-IB, 12-IA, 14-IC, 16-DB, 17-DA, and 20-AVI).

The fingerprints of the obtained RRD and SRD are shown in Figures 1 and 2. The results in Tables 4 and 5 showed that the similarity of the fingerprints of raw and salt-processed RD was above 0.900, respectively, and a total of 25 peaks were obtained.  Figure 3 shows that compared with the results of the reference solution, 10 components were identified, namely, peak 6-LA; peak 8-CA; peak 9-CaA; peak 10-LN; peak 11-IB; peak 12-IA; peak 14-IC; peak 16-DB; peak 17-DA; peak 20-AVI.

### 4.3. Result of Contents Determination

As shown in the results from Tables 6 and 7, the LA content in different batches of RRD sample was 1.32∼1.92%, CA was 0.34∼0.55%, CaA was 0.01∼0.03%, LN was 0.24∼0.51%, IB was 0.03∼0.11%, IA was 0.44∼0.60%, IC was 0.21∼0.56%, DB was 0.13∼0.16%, DA was 0.23∼0.53%, and AVI was 4.92∼8.86%. The LA content in SRD sample was 1.49∼2.72%, CA was 0.27∼5.00%, CaA was 0.02∼0.11%, LN was 0.05∼0.43%, IB was 0.01∼0.06%, IA was 0.28∼0.58%, IC was 0.15∼0.32%, DB was 0.00∼0.12%, DA was 0.29∼0.42%, and AVI was 6.19∼9.35%.

**Figure 4:**
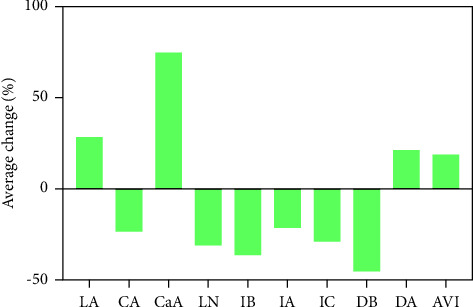
Changes in content of RD after processing with salt.

After processing, the contents of 10 components in the corresponding batches of raw and salted-processed RD were significantly different. As shown in Figure 4, the contents of LA, CaA, DA, and AVI in SRD were higher than RRD, the average change rates were 28.80%, 74.78%, 21.38%, and 18.99%, respectively, and the contents of CA, LN, IB, IA, IC, and DB in SRD were lower than those in RRD, with an average change rate of −23.28%, −30.96%, −36.30%, −21.49%, −29.49%, and −45.37%, respectively. This was due to the change in content caused by processing with salt, and it was speculated that the content of phenolic acids and iridoid glycosides in RD might be reduced due to the conversion and degradation of the components after heating.

### 4.4. Analysis of the Difference between Raw and Salt-Processed RD

#### 4.4.1. PCA

**Figure 5:**
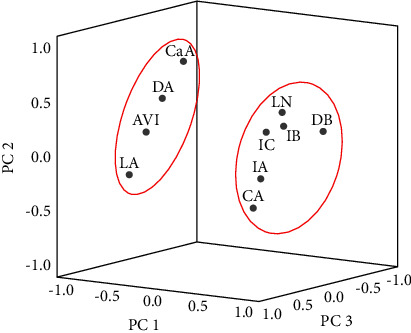
Three-dimensional map of the compound. (Note: “PC” stands for principal component).

In this experiment, SPSS 25.0 was used to perform PCA on 26 samples. The results are shown in Table 8, the principal components were extracted with the eigenvalue *λ* > 1, *λ*_1_ = 4.324, the contribution rate was 43.237%, *λ*_2_ = 1.512, the contribution rate was 15.122%, *λ*_3_ = 1.395, the contribution rate was 13.950%, and the contribution rate of the first principal component was the largest, indicating that it contains the most information. When the number of principal components was 3, the cumulative contribution rate reached 72.310%, and it indicated that the first three principal components could represent most of the information data about the difference between the raw and salt-processed RD. The first, second, and third principal components were used as the coordinate system, and the three-dimensional map of each compound was obtained by projection. As shown in Figure 5, the 10 compounds were divided into 2 categories, one of which was LA, CaA, DA, and AVI and the other type was CA, LN, IB, IA, IC, and DB. This result was consistent with the change law of the content before and after processing with salt, the former was the components whose content increased after processing with salt, and the latter was the components whose content decreased.

The component loading matrix can explain the contribution rate of each variable to the principal component. The greater the absolute value of the compound loading, the greater the contribution to the principal component, indicating that it is more important in the quality control of decoction pieces. According to the data in Table 9, by comparing the absolute value of the load of the compound in the “most informative” first principal component, it could be seen that the importance of the above 10 compounds in the quality control of raw and salt-processed RD was LA > DB > IA > IC > IB > LN > CA > DA > AVI > CaA.

#### 4.4.2. Fisher Discriminant Analysis

FDA is one of the methods of discriminant analysis. It uses the idea of variance analysis to project points in a high-dimensional space to a low-dimensional space to construct one or more linear discriminant functions in different-dimensional spaces. The contents of 10 compounds in RRD and SRD were selected as variables to generate FDA. The results of the coefficients are shown in Table 10, *X*_1_, *X*_2_,…, *X*_10_ in the function expressions were used to represent the normalized data of the content of each compound respectively, and the function expressions were shown as follows:

(1) $$\begin{array}{c} \text{RRD}=323.095X_{1}+490.050X_{2}+2368.596X_{3}-167.278X_{4}+1648.097X_{5}+35.132X_{6}-113.313X_{7}+2301.225X_{8}-242.518X_{9}-39.507X_{10}-406.748,\\ \text{SRD}=274.028X_{1}+378.002X_{2}+1964.572X_{3}-153.714X_{4}+1382.986X_{5}+17.266X_{6}-102.903X_{7}+1912.431X_{8}-35.987X_{9}-28.381X_{10}-342.073. \end{array}$$

**Figure 6:**
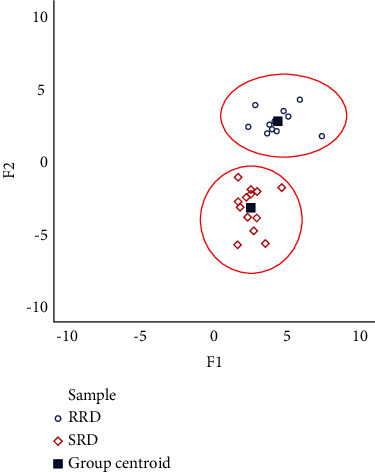
FDA graph of raw and salt-processed RD.

The above discriminant functions were used to back-substitute the classification. As shown in Figure 6, the samples of raw and salt-processed RD could be well differentiated in the discriminant analysis scatterplot. At the same time, the discriminants of RRD and SRD were consistent with the actual, and the accuracy rates were both 100%.

## 5. Discussion

The HPLC fingerprints of RD before and after processing with salt were established. There were 25 common peaks in the fingerprints, and the contents of 10 components were determined. The results showed that the contents of LA, CaA, DA, and AVI increased, while the contents of CA, LN, IB, IA, IC, and DB decreased after processing with salt. No new or disappeared components were found in the RD before and after processing with salt, and it was speculated that there might have been intercomponent transformations. For example, the increase in LA content may have been caused by the addition of the -COOH group to LN, which was also consistent with the decrease of LN and organic acids (CA, IB, IA, and IC). The conversion between DA and DB led to an increase in DA content. The study of the transformation between these components was also an important part of the processing mechanism of TCM. Whether the change in composition after processing with salt was caused by heating or salt processing during frying has not been systematically studied in this section. Subsequent systematic studies will be carried out on the effect of excipient salt on the composition.

Chemometrics, also known as chemical statistics, is a branch of chemistry that combines mathematics, statistics, computer science, and chemistry, which the most important feature of chemistry is the introduction of multivariate analysis methods into chemical research and the multivariate processing and analysis of chemical measurement data. Chemometrics include measurement tests, chemical pattern recognition, regression analysis, and multivariate correction [15, 16]. PCA is an unsupervised pattern recognition analysis which uses the idea of reducing the dimension of the data matrix to convert the original indicators into several comprehensive indicators through a linear transformation under the premise of losing a small amount of information, so as to simplify datasets and visualize differences between samples [17]. Since there is no human involvement in the analysis process, and the calculation model is based on the state of the original variables, PCA is very helpful in reflecting and expressing the overall situation of the variables under analysis and the total control of variables by the researcher, which helps to identify and eliminate problematic samples and abnormal variables, thus improving the accuracy and precision of the mathematical analysis model [18–20]. The use of PCA can simplify complex multivariate data systems, and more studies have reported its use in the study of TCM. Many studies have been reported on its use in the study of Chinese medicine and natural drugs [21–23]. In this study, the results of PCA showed that the order of influence of 10 components in the classification of raw and salt-processed RD was LA > DB > IA > IC > IB > LN > CA > DA > AVI > CaA.

Discriminant analysis is a supervised classification technique belonging to chemometrics, which classifies certain objects studied based on certain observed indicators. In the quality control experiment of TCM, discriminant analysis can establish a discriminant based on the observation data of a batch of known samples of various types and then classify the unknown types of samples. FDA is a common method in discriminant analysis, which is generally used to discriminate two kinds of quantitative data [24]. It uses the idea of one-dimensional ANOVA to reduce the sample points in the n-dimensional space to one-dimensional data by means of linear functions and then classifies the sample points to be judged into different categories according to the distance between samples. FDA can make the differences between sample points in the same category as small as possible and make the differences between sample points in different categories as large as possible, thus effectively improving the discriminant efficiency [25, 26]. This analytical approach of the FDA was applied in this paper, and the results showed that the model and algorithm given in the paper were effective and useful for the classification of raw and salt-processed RD.

## 6. Conclusion

In this study, the content of 10 chemical components before and after processing with salt of RD was determined by HPLC-DAD and was sorted through PCA to rank the importance of each chemical component. At the same time, the samples were classified and verified by the FDA. The results showed that the components in RD after processing with salt had internal transformation, the contents of LA, CaA, DA, and AVI increased, and the contents of CA, LN, IB, IA, IC, and DB decreased. In the classification of raw and salt-processed RD, the order of importance of each chemical component was LA > DB > IA > IC > IB > LN > CA > DA > AVI > CaA. These components could be used as differential components to identify raw and salt-processed RD. This study provides a comprehensive and quantitative chemical pattern recognition and quality evaluation method for the identification of TCM before and after processing. This method could also provide a scientific basis for further research on the spectrum-effect relationship and mechanism of action.

## Figures and Tables

**Table 1:** The sample information of raw and salt-processed RD.

No	Origin	Batch number	No	Origin	Batch number
R1	Sichuan	S201908	S1	Sichuan	Y201908
R2	Sichuan	S201903	S2	Sichuan	Y201903
R3	Yunnan	S201911-1	S3	Yunnan	Y201911-1
R4	Yunnan	S201911-2	S4	Yunnan	Y201911-2
R5	Sichuan	S20190157	S5	Sichuan	Y20190157
R6	Yunnan	S20190417	S6	Yunnan	Y20190417
R7	Sichuan	S20190416	S7	Sichuan	Y20190416
R8	Hunan	S20051514	S8	Hunan	Y20051514
R9	Hunan	S20051515	S9	Hunan	Y20051515
R10	Hunan	S20051516	S10	Hunan	Y20051516
R11	Guizhou	S2005054	S11	Guizhou	Y2005054
R12	Hubei	S201910	S12	Hubei	Y201910
R13	Shandong	S202004211	S13	Shandong	Y202004211

**Table 2:** The results of linear relation (*n* = 10).

Component	Regression equation	*r*	Linear range (*μ*g/mL)	LOD (*μ*g/mL)	LOQ (*μ*g/mL)
LA	*y* = 0.0658*x* − 0.1336	0.9997	47.68∼953.54	0.15	0.50
CA	*y* = 0.3312*x* − 0.0990	0.9997	23.86∼477.26	0.15	0.50
CaA	*y* = 0.6283*x* + 0.3508	0.9997	21.04∼420.73	0.12	0.35
LN	*y* = 0.0992*x* + 0.1302	0.9997	59.25∼1185.03	0.12	0.35
IB	*y* = 0.3718*x* + 0.2226	0.9997	30.14∼602.70	0.15	0.51
IA	*y* = 0.4154*x* + 0.0929	0.9997	41.50∼830.06	0.10	0.35
IC	*y* = 0.4199*x* − 0.0743	0.9998	30.14∼602.70	0.10	0.35
DB	*y* = 0.0873*x* + 0.0072	0.9997	2.01∼40.22	0.10	0.35
DA	*y* = 0.1289*x* − 0.0052	0.9997	5.54∼110.85	0.11	0.35
AVI	*y* = 0.0806*x* + 0.0581	0.9999	97.60∼1952.01	0.15	0.50

**Table 3:** The results of sample recovery rate (*n* = 6).

Sample	Component	Initial amount (mg ± SD)	Injection amount (mg)	Total amount (mg ± SD)	Recovery rate (%±SD)	RSD (%)
RRD	LA	4.38 ± 0.01	4.77	9.17 ± 0.12	100.34 ± 2.40	2.39
	CA	1.24 ± 0.00	1.19	2.43 ± 0.01	99.46 ± 2.95	2.96
	CaA	0.03 ± 0.00	0.03	0.06 ± 0.00	100.62 ± 4.73	4.70
	LN	0.99 ± 0.00	0.95	1.96 ± 0.03	101.88 ± 3.02	3.92
	IB	0.14 ± 0.00	0.15	0.29 ± 0.00	103.86 ± 2.81	2.70
	IA	1.37 ± 0.00	1.66	3.06 ± 0.03	101.96 ± 1.78	1.75
	IC	0.74 ± 0.00	0.60	1.34 ± 0.01	99.10 ± 2.19	2.21
	DB	0.34 ± 0.00	0.32	0.60 ± 0.01	99.36 ± 3.24	3.26
	DA	0.74 ± 0.00	0.83	1.60 ± 0.01	102.82 ± 1.88	1.83
	AVI	17.33 ± 0.03	15.61	33.39 ± 0.28	102.91 ± 1.72	1.67

SRD	LA	5.59 ± 0.01	4.77	10.47 ± 0.09	102.31 ± 1.94	1.89
	CA	1.28 ± 0.00	1.19	2.49 ± 0.03	102.34 ± 2.74	2.68
	CaA	0.11 ± 0.00	0.11	0.22 ± 0.00	102.92 ± 2.31	2.25
	LN	0.91 ± 0.00	0.95	1.89 ± 0.02	103.70 ± 1.59	1.54
	IB	0.09 ± 0.00	0.09	0.18 ± 0.00	99.06 ± 3.22	3.25
	IA	1.26 ± 0.00	1.66	2.93 ± 0.05	100.56 ± 2.94	2.92
	IC	0.73 ± 0.00	0.60	1.34 ± 0.02	101.01 ± 2.70	2.66
	DB	0.01 ± 0.00	0.01	0.02 ± 0.00	99.30 ± 3.52	3.54
	DA	0.81 ± 0.00	0.83	1.66 ± 0.03	102.32 ± 3.05	2.98
	AVI	18.26 ± 0.01	19.52	37.96 ± 0.46	102.47 ± 2.79	2.72

**Table 4:** The similarity results of 13 batches of RRD.

No	Similarity
R1	1.000
R2	0.912
R3	0.925
R4	0.957
R5	0.923
R6	0.974
R7	0.916
R8	0.947
R9	0.918
R10	0.912
R11	0.916
R12	0.936
R13	0.970

**Table 5:** The similarity results of 13 batches of SRD.

No	Similarity
S1	0.921
S2	0.910
S3	0.927
S4	0.936
S5	0.973
S6	0.959
S7	0.987
S8	0.958
S9	0.972
S10	0.926
S11	0.974
S12	0.934
S13	0.923

**Table 6:** Contents of 10 components in RRD (%, *n* = 6).

No	LA	CA	CaA	LN	IB	IA	IC	DB	DA	AVI
R1	1.92	0.55	0.01	0.43	0.06	0.60	0.32	0.15	0.33	7.59
R2	1.48	0.52	0.03	0.38	0.05	0.56	0.29	0.13	0.29	6.19
R3	1.49	0.52	0.02	0.24	0.05	0.57	0.30	0.13	0.29	6.23
R4	1.47	0.52	0.02	0.24	0.05	0.56	0.29	0.13	0.28	6.17
R5	1.44	0.34	0.03	0.51	0.05	0.44	0.36	0.16	0.25	5.69
R6	1.32	0.40	0.03	0.41	0.11	0.56	0.54	0.14	0.30	4.92
R7	1.92	0.55	0.03	0.46	0.03	0.60	0.56	0.15	0.23	8.86
R8	1.48	0.52	0.03	0.31	0.04	0.56	0.29	0.13	0.28	5.79
R9	1.49	0.52	0.03	0.31	0.04	0.57	0.21	0.13	0.28	5.79
R10	1.60	0.55	0.03	0.43	0.04	0.60	0.32	0.15	0.28	5.79
R11	1.48	0.52	0.02	0.31	0.04	0.56	0.29	0.13	0.28	5.79
R12	1.32	0.52	0.03	0.41	0.11	0.57	0.54	0.13	0.30	6.23
R13	1.47	0.52	0.02	0.46	0.05	0.56	0.29	0.13	0.23	6.17
X	1.53	0.50	0.02	0.38	0.06	0.56	0.35	0.14	0.28	6.25
SD	0.19	0.06	0.01	0.09	0.03	0.04	0.11	0.01	0.03	0.98

**Table 7:** Contents of 10 components in SRD (%, *n* = 6).

No	LA	CA	CaA	LN	IB	IA	IC	DB	DA	AVI
S1	2.33	0.53	0.04	0.38	0.04	0.53	0.30	0.01	0.34	7.62
S2	2.29	0.51	0.03	0.24	0.04	0.55	0.29	0.01	0.42	7.17
S3	2.72	0.27	0.03	0.06	0.01	0.29	0.15	0.01	0.30	7.22
S4	2.31	0.45	0.02	0.05	0.04	0.51	0.29	0.00	0.31	7.47
S5	1.71	0.30	0.11	0.32	0.03	0.39	0.26	0.09	0.41	8.11
S6	1.64	0.31	0.05	0.38	0.06	0.51	0.28	0.12	0.35	6.66
S7	2.22	0.30	0.04	0.43	0.02	0.58	0.32	0.08	0.36	7.59
S8	1.60	0.37	0.03	0.24	0.03	0.39	0.21	0.12	0.35	6.19
S9	1.58	0.37	0.03	0.24	0.03	0.38	0.28	0.12	0.35	6.23
S10	1.92	0.37	0.04	0.31	0.03	0.39	0.21	0.12	0.33	7.59
S11	1.58	0.37	0.03	0.24	0.03	0.38	0.21	0.12	0.29	6.19
S12	1.49	0.53	0.06	0.24	0.05	0.56	0.30	0.12	0.30	9.35
S13	2.22	0.30	0.04	0.24	0.02	0.28	0.17	0.08	0.31	9.23
X	1.97	0.39	0.04	0.26	0.04	0.44	0.25	0.08	0.34	7.43
SD	0.40	0.09	0.02	0.11	0.01	0.10	0.06	0.05	0.04	1.03

**Table 8:** Principal component eigenvalue and contribution rate.

Principal component	*λ*	Contribution rate (%)	Cumulative contribution rate (%)
1	4.324	43.237	43.237
2	1.512	15.122	58.359
3	1.395	13.950	72.310
4	0.946	9.456	81.766
5	0.718	7.177	88.943
6	0.529	5.291	94.234
7	0.257	2.566	96.800
8	0.166	1.661	98.461
9	0.122	1.219	99.681
10	0.032	0.319	100.000

**Table 9:** Principal component loading matrix.

Compound	Principal component		
	1	2	3
LA	−0.767	−0.227	0.450
CA	0.628	−0.389	0.487
CaA	−0.439	0.795	0.062
LN	0.653	0.432	0.067
IB	0.696	0.315	0.103
IA	0.737	−0.103	0.525
IC	0.713	0.305	0.401
DB	0.754	0.194	−0.435
DA	−0.575	0.461	0.202
AVI	−0.535	0.197	0.519

**Table 10:** Fisher linear discriminant function coefficients of raw and salt-processed RD.

Compound	RRD	SRD
LA	323.095	274.028
CA	490.050	378.002
CaA	2368.596	1964.572
LN	−167.278	−153.714
IB	1648.097	1382.986
IA	35.132	17.266
IC	−113.313	−102.903
DB	2301.225	1912.431
DA	−242.518	−35.987
AVI	−39.507	−28.381
Constant	−406.748	−342.073

## Data Availability

Data available on request from the authors. The data that support the findings of this study are available from the corresponding author upon reasonable request.

## Abbreviations

RD: Radix Dipsaci
LN: Loganin
AVI: Asperosaponin VI
CaA: Caffeic acid
DA: Dipsanoside A
DB: Dipsanoside B
CA: Chlorogenic acid
LA: Loganic acid
IA: Isochlorogenic acid A
IB: Isochlorogenic acid B
IC: Isochlorogenic acid C
PCA: Principal component analysis
FDA: Fisher discriminant analysis
RRD: Raw Radix Dipsaci
SRD: Salt-processed Radix Dipsaci
TCM: Traditional Chinese medicine
LOD: Limit of detection
LOQ: Limit of quantification
RRT: Relative retention time
RPA: Relative peak area
PC: Principal component.
